# Lycopene ameliorates locomotor activity and urinary frequency induced by pelvic venous congestion in rats

**DOI:** 10.1515/med-2023-0638

**Published:** 2023-02-25

**Authors:** Jinchai Zhao, Wei Chen, Jian Liu

**Affiliations:** Department of Gynecology, The Second Hospital of Hebei Medical University, Shijiazhuang City, Hebei Province, 050000, China; Department of Pharmacy, The Second Hospital of Hebei Medical University, Shijiazhuang City, Hebei Province, 050000, China; Department of Pharmacy, The Second Hospital of Hebei Medical University, 215 West Heping Road, Xinhua District,, Shijiazhuang City, Hebei Province, 050000, China

**Keywords:** lycopene, locomotor activity, urinary frequency, NF-κB, pelvic venous congestion

## Abstract

Decreased locomotor activity and altered urinary frequency are induced by bilateral common iliac vein ligation in rats. As a carotenoid, lycopene has a strong anti-oxidative function. This research investigated the function of lycopene in the pelvic venous congestion (PC) rat model and the underlying molecular mechanism. Lycopene and olive oil were administered intragastrically on a daily basis for 4 weeks after successful modeling. Locomotor activity, voiding behavior, and continuous cystometry were analyzed. The levels of 8-hydroxy-2′-deoxyguanosine (8-OHdG), nitrate and nitrite (NO_*x*_), and creatinine in the urine were measured. Gene expression in the bladder wall was analyzed by quantitative reverse transcription polymerase chain reaction, enzyme-linked immunosorbent assay, and Western blot. Locomotor activity, single voided volume, the interval between the bladder contractions, and urinary NO_*x*_/cre ratio were all decreased in rats with PC, while the frequency of urination, urinary 8-OHdG/cre ratio, inflammatory responses, and nuclear factor-κB (NF-κB) signal activity were all increased. Lycopene treatment increased locomotor activity, decreased frequency of urination, elevated urinary NO_*x*_ level, and decreased urinary 8-OHdG level in the PC rat model. Lycopene also inhibited PC-enhanced pro-inflammatory mediator expression and NF‐κB signaling pathway activity. In conclusion, lycopene treatment ameliorates PC-induced phenotypes and shows an anti-inflammatory effect in the PC rat model.

## 1 Introduction

Pelvic congestion syndrome is caused by unilateral or bilateral ovarian vein incompetence [1,2]. The pathogenesis of pelvic congestion syndrome induces pelvic organ dysfunction, dysmenorrhea, dyspareunia, urinary urgency, irritable bladder, chronic pelvic pain, and varicose veins and vulval varices [3,4]. Pelvic venous congestion (PC) contributes to chronic pelvic pain [5]. The main cause of PC is the gonadal vein valves, and its main symptoms are pelvic venous engorgement and gonadal vein reflux [6].

In spontaneously hypertensive rats, prostate blood flow, bladder capacity, and voiding volume are all decreased [7,8]. The overactivity of detrusor-caused voiding frequency is observed in rats with atherosclerosis-induced chronic bladder ischemia [9]. Studies have demonstrated that decreased locomotor activity and altered urinary frequency are induced in the PC rat model [10]. Surgically altered rats exhibited reduced bladder blood flow by ∼20% compared to intact bladder flow [10]. Thus, PC may be related to pelvic ischemia and other urinary tract diseases [11].

Lycopene is widely distributed in different kinds of fruits. Based on its special conjugated double bonds, lycopene has a strong anti-oxidative capacity [12]. In rats with pentylenetetrazole-induced epileptic seizures and memory impairment, lycopene supplementation displayed anti-epileptic activity by inhibiting the inducible nitric oxide synthase (iNOS) pathway [13]. Lycopene can attenuate chronic pelvic pain syndrome by inhibiting inflammation and oxidative stress via the nuclear factor-κB (NF-κB) pathway [14]. In this study, we investigated lycopene function in the PC rat model and the underlying molecular mechanism.

## 2 Materials and methods

### 2.1 Animals

Animal studies were approved by the Institutional Animal Care and Use Committee of the Second Hospital of Hebei Medical University. In this study, female Sprague–Dawley rats weighing 200–230 g were used. Rats were anesthetized with 2% isoflurane. After lower abdomen dissection, the bilateral uterine veins were ligated with the uterine artery, and the uterine horns near the ovaries and the bilateral common iliac veins were ligated with metal clips. After venous ligation, the distal common iliac vein was dilated. Antibiotics (30 mg of ampicillin) were administered subcutaneously to all the animals after closing the abdomen. The rats then recovered in the dam for 2 h post-surgery.

In the sham group, the bilateral common iliac veins were dissected free of the common iliac arteries. Rats with PC were randomized into four groups: PC group, PC + 5 mg/kg/day lycopene (PC + Lyc5) group, PC + 10 mg/kg/day lycopene (PC + Lyc10) group, and PC + 20 mg/kg/day lycopene (PC + Lyc20) group. Lycopene (purity ≥98%; Solarbio, Wuhan, China) was dissolved in olive oil. Lycopene and olive oil were administered intragastrically on a daily basis for 4 weeks after successful modeling. The rats in the sham group and the PC groups received the same volume of olive oil as the lycopene-treated groups.

After the 4-week treatment, locomotor activity and urinary voiding tests were performed, and spontaneously voided urine was collected. The continuous cystometric parameters, 8-hydroxy-2′-deoxyguanosine (8-OHdG), nitrate and nitrite (NO_*x*_), and creatinine levels were analyzed. The rats were sacrificed, and the bladders were collected and cut into halves longitudinally. Half of the bladder was homogenized in 50 mM Tris–HCl pH 7.4 (1/10, w/v) and stored at −80℃ for enzyme-linked immunosorbent assay (ELISA), and the other half was mixed in groups and subjected to four replicates of polymerase chain reaction (PCR) or Western blot experiments.

### 2.2 Locomotor activity

The rats were housed individually, and the locomotor activity was measured by the infrared sensor and digital counter (NS-ASS01; Neuroscience, Inc., Tokyo, Japan). The sum of all movements between 8:00 p.m. and 1:00 a.m. was calculated as locomotor activity.

### 2.3 Voiding behavior

The rats were housed individually for 24 h. During the assessment, integrating urine weight, voiding volumes, and voiding times were measured at 1-min intervals through a computer with a camera (Mijia, China).

### 2.4 Continuous cystometry

After being anesthetized, rats were kept in a restraining cage. A polyethylene catheter (NS-ASS01; Neuroscience, Inc.) connected with a pressure transducer and infusion pump was transurethrally placed in the bladder. About 0.05 mL of saline was pumped into the bladder within a minute and continued for at least 90 min. Bladder activity was evaluated.

### 2.5 8-OHdG, NO_*x*_, and creatinine measurements

Spontaneously voided urine was gathered for measurement. The levels of creatinine and 8-OHdG were analyzed by commercial ELISA kits (Trevigen, Gaithersburg, MD, USA). NO_*x*_ was evaluated through the Griess method by a high-performance liquid chromatography system.

### 2.6 IL-1β, IL-6, and iNOS measurements

In the bladder, the iNOS, interleukin 1β (IL-1β) and interleukin 6 (IL-6) levels were analyzed through corresponding ELISA kits (MultiSciences Biotech, China).

### 2.7 Quantitative reverse transcription polymerase chain reaction (qRT-PCR)

Total ribonucleic acid (RNA) was extracted from the bladder tissue with an RNeasy Mini Kit (Qiagen, Hilden, Germany). After being treated with DNase I to avoid genomic contamination, 1 μg of RNA was used for the synthesis of complementary deoxyribonucleic acid with the SuperScript™ II Reverse Transcriptase (ThermoFisher Scientific). Real-time PCR was executed by the SYBR Green Real-Time PCR Master Mixes (ThermoFisher, Waltham, MA, USA).

*IL-1β* F: CCACCTCCAGGGACAGGATA

*IL-1β* R: AACACGCAGGACAGGTACAG;

*IL-6* F: CCGTTTCTACCTGGAGTTTG

*IL-6* R: GTTTGCCGAGTAGACCTCAT;

*iNOS* F: GATCAATAACCTGAAGCCCG

*iNOS* R: GCCCTTTTTTGCTCCATAGG;

*GAPDH* F: GTGCCAGCCTCGTCTCATAG

*GAPDH* R: CTTTGTCACAAGAGAAGGCAG.

### 2.8 Western blot

Tissues were lysed by a radioimmunoprecipitation assay buffer (Beyotime, Nantong, China). The Western blot was performed using the standard method. Antibodies used in this experiment included anti-p65 (Cell Signaling Technology, Danvers, MA, USA), anti-p-p65 (Cell Signaling Technology), anti-iNOS (Santa Cruz, Biotechnology, Santa Cruz, CA, USA), and anti-β-actin (Santa Cruz).

### 2.9 Statistical analysis

Statistical analysis was performed by the GraphPad PRISM 6.0 software. Data were presented as mean ± standard deviation (SD). One-way analysis of variance (ANOVA) followed by Dunn’s multiple comparisons test was used to calculate the differences between each group.

## 3 Results

### 3.1 Lycopene ameliorates decreased locomotor activity in rats with PC

**Figure 1:**
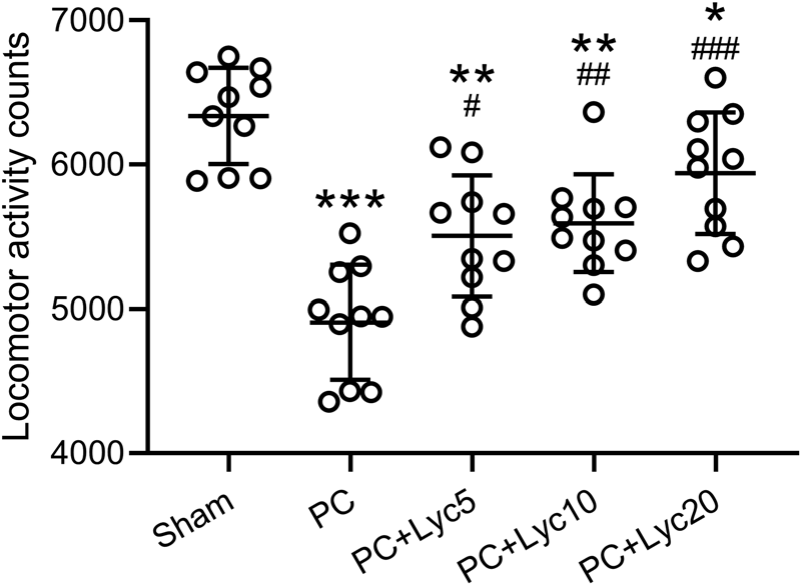
Effects of lycopene on locomotor activity of rats with PC. Locomotor activity during the dark period was compared among different groups. *N* = 10 for each group. Mean ± SD with all data presented. One-way ANOVA followed by Dunn’s multiple comparisons test. ^#^ *p* < 0.05, ^##^ *p* < 0.01, ^###^ *p* < 0.001 compared to the PC group. ^*^ *p* < 0.05, ^**^ *p* < 0.01, ^***^ *p* < 0.001 compared to the sham group.

As shown in Figure 1, the PC group showed remarkably lower locomotor activity than the sham group. Lycopene treatment at doses of 5, 10, and 20 mg/kg/day increased locomotor activity in a dose-dependent manner (Figure 1). The representative actograms of locomotor activities among different groups are shown in Figure S1.

### 3.2 Lycopene ameliorates increased urinary frequency in rats with PC

**Figure 2:**
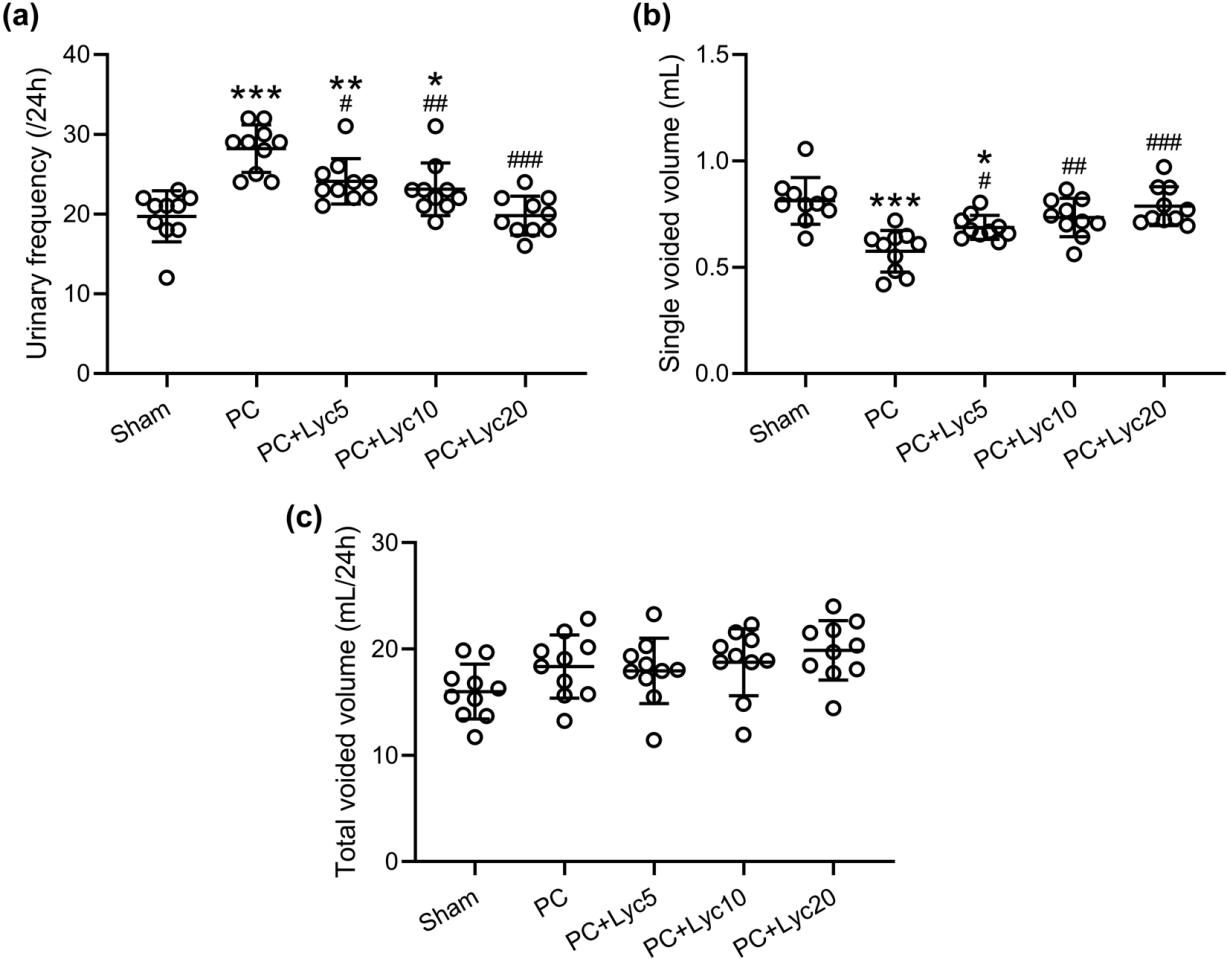
Effects of lycopene on the voiding behavior of rats with PC. The 24-h frequency of urination (a), single-voided volume during the light and dark periods (b), and total voided urine volume at 24 h (c) were compared. *N* = 10 for each group. Mean ± SD with all data presented. One-way ANOVA followed by Dunn’s multiple comparisons test. ^#^ *p* < 0.05, ^##^ *p* < 0.01, ^###^ *p* < 0.001 compared to the PC group. ^*^ *p* < 0.05, ^**^ *p* < 0.01, ^***^ *p* < 0.001 compared to the sham group.

As shown in Figure 2a, the PC group exhibited a significantly higher frequency of urination than the sham group, the PC + Lyc5 group, the PC + Lyc10 group, and the PC + Lyc20 group. The PC group showed markedly lower single-voided volume than the other groups (Figure 2b). However, the total voided volume was not influenced (Figure 2c). Lycopene treatment at doses of 5, 10, and 20 mg/kg/day attenuated the frequency of urination and increased the single voided volume in rats with PC.

### 3.3 Lycopene ameliorates shortened interval between bladder contractions in rats with PC

**Figure 3:**
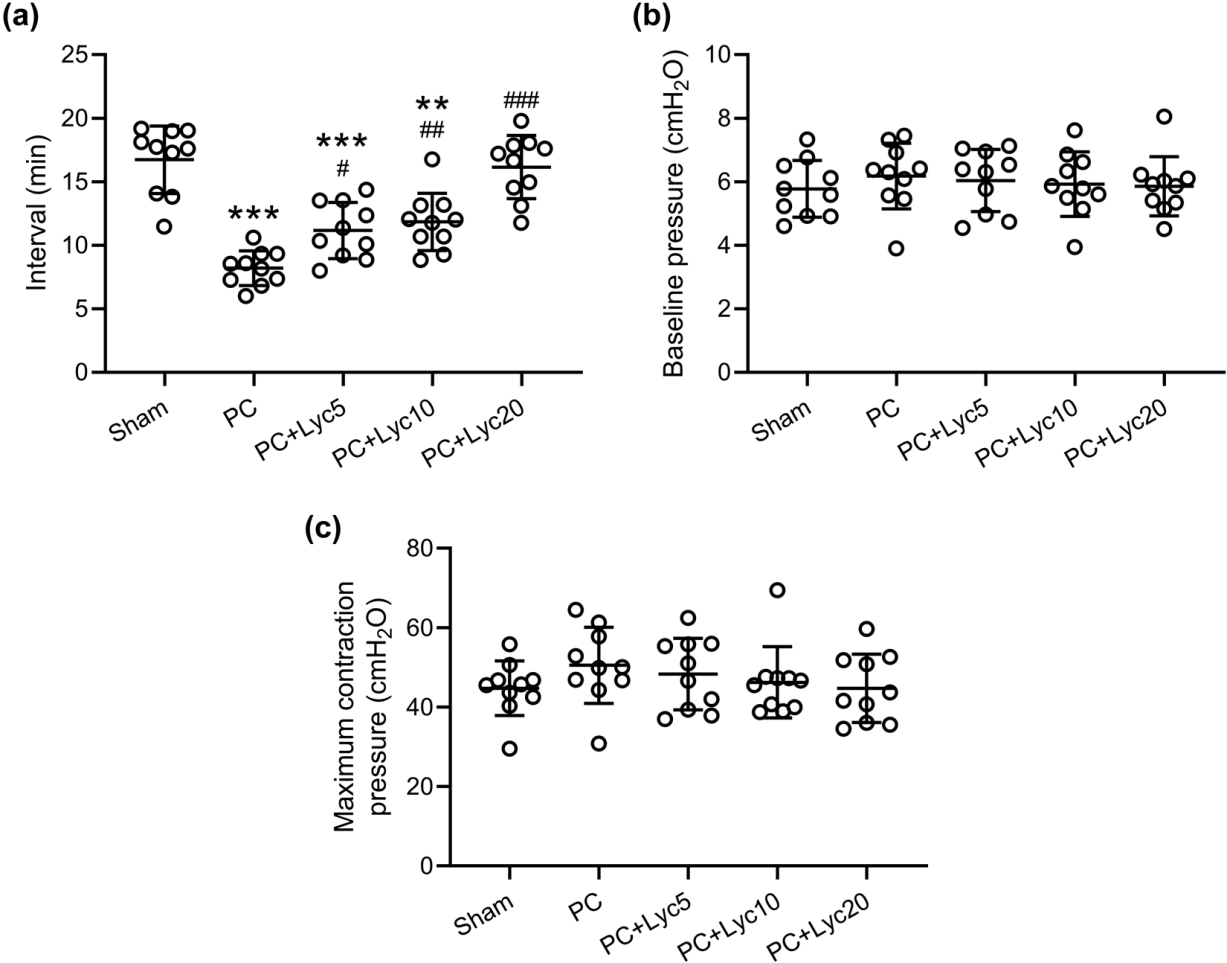
Effects of lycopene on the continuous cystometric parameters. The interval between the bladder contractions (a), the bladder baseline pressure (b), and the maximum bladder contraction pressure (c) were recorded. *N* = 10 for each group. Mean ± SD with all data presented. A one-way ANOVA followed by Dunn’s multiple comparisons test. ^#^ *p* < 0.05, ^##^ *p* < 0.01, ^###^ *p* < 0.001 compared to the PC group. ^**^ *p* < 0.01, ^***^ *p* < 0.001 compared to the sham group.

The PC group showed a shorter interval between bladder contractions than the other groups, while the lycopene treatment at doses of 5, 10, and 20 mg/kg/day could increase the interval in rats with PC (Figure 3a). Maximum bladder contraction pressure and bladder baseline pressure in these groups showed no significant difference (Figure 3b and c).

### 3.4 Lycopene ameliorates altered urinary NO_*x*_ and 8-OHdG levels in rats with PC

**Figure 4:**
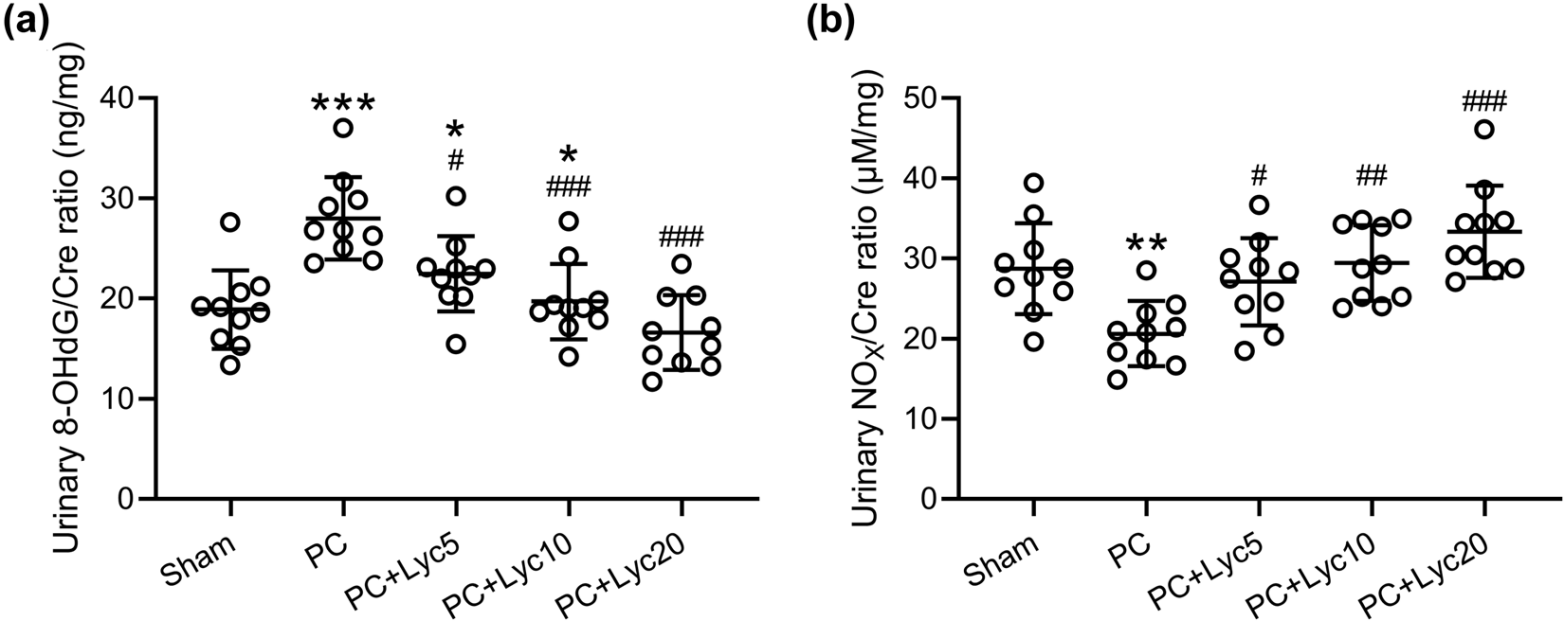
Effects of lycopene on 8-OHdG and NO_*x*_ levels. Comparisons of the urinary 8-OHdG (a) and NO_*x*_ (b) levels corrected for creatinine among different groups. *N* = 10 for each group. Mean ± SD with all data presented. One-way ANOVA followed by Dunn’s multiple comparisons test. ^#^ *p* < 0.05, ^##^ *p* < 0.01, ^###^ *p* < 0.001 compared to the PC group. ^*^ *p* < 0.05, ^***^ *p* < 0.001 compared to the sham group.

In the PC group, the 8-OHdG/creatinine ratio was significantly higher than in the other groups (Figure 4a). In the PC group, the urinary NO_*x*_/creatinine ratio was lower than in the other groups (Figure 4b). Lycopene treatment at doses of 5, 10, and 20 mg/kg/day successfully decreased the 8-OHdG/creatinine ratio while increasing the NO_*x*_/creatinine ratio in rats with PC.

### 3.5 Lycopene ameliorates enhanced inflammatory responses in the bladder of rats with PC

**Figure 5:**
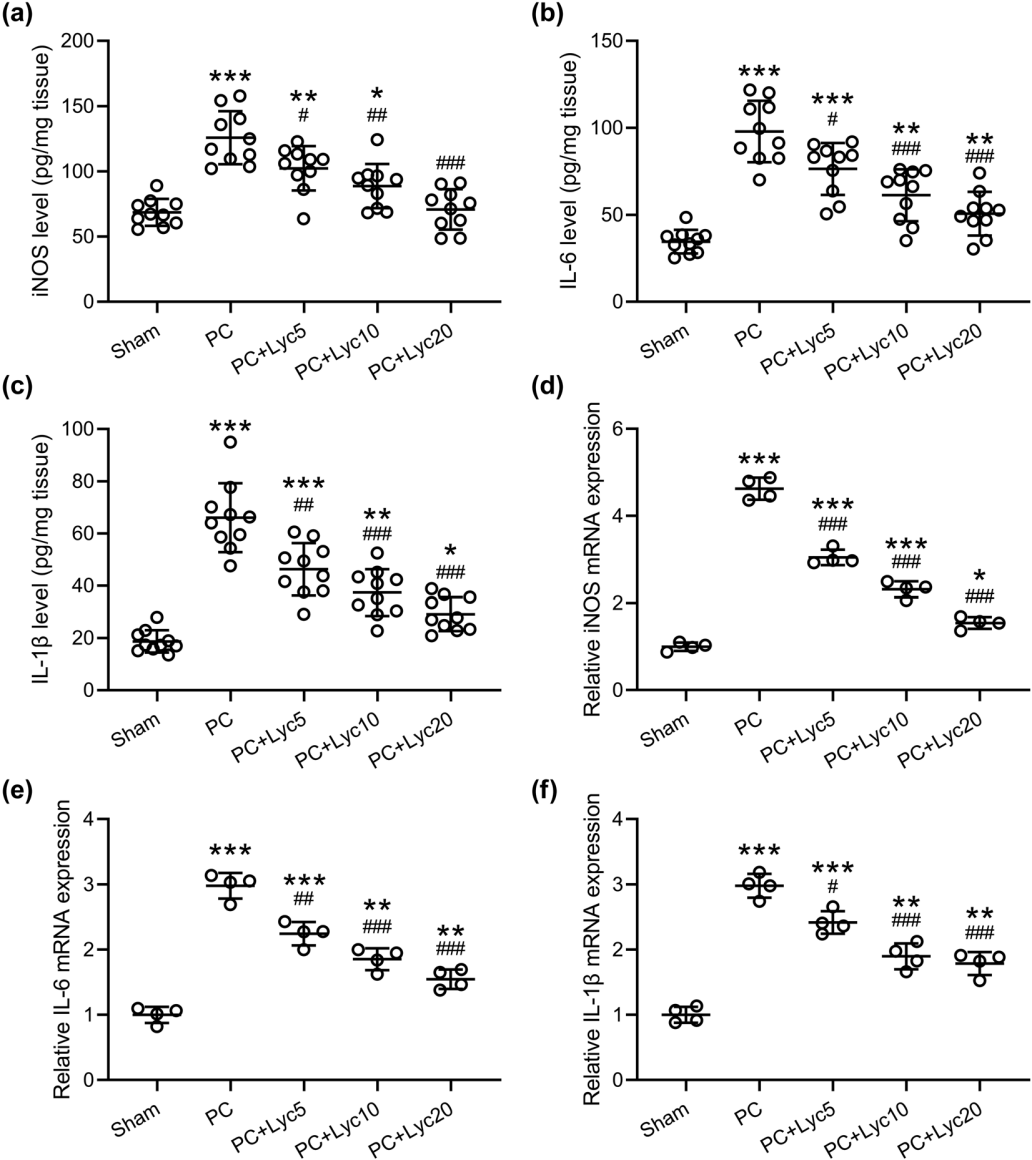
Effects of lycopene on inflammatory response in the bladder of rats with PC. iNOS (a), IL-6 (b), and IL-1β (c) levels in the bladder among different groups were measured by ELISA. *N* = 10 for each group. The mRNA levels of iNOS (d), IL-6 (e), and IL-1β (f) in the bladder were measured by qRT-PCR. *N* = 4 from ten mixed tissues for each group. Mean ± SD with all data presented. One-way ANOVA followed by Dunn’s multiple comparisons test. ^#^ *p* < 0.05, ^##^ *p* < 0.01, ^###^ *p* < 0.001 compared to the PC group. ^*^ *p* < 0.05, ^**^ *p* < 0.01, ^***^ *p* < 0.001 compared to the sham group.

Based on the ELISA results, iNOS, IL-1β, and IL-6 levels in the PC group were significantly higher than in the sham group, the PC + Lyc5 group, the PC + Lyc10 group, and the PC + Lyc20 group (Figure 5a–c). iNOS, IL-1β, and IL-6 mRNA levels showed the same tendency (Figure 5d–f). Lycopene treatment at doses of 5, 10, and 20 mg/kg/day successfully attenuated the inflammatory responses in rats with PC, as evidenced by the decreased levels of iNOS, IL-1β, and IL-6.

### 3.6 Lycopene ameliorates enhanced expressions of iNOS and NF-κB in the bladder of rats with PC

**Figure 6:**
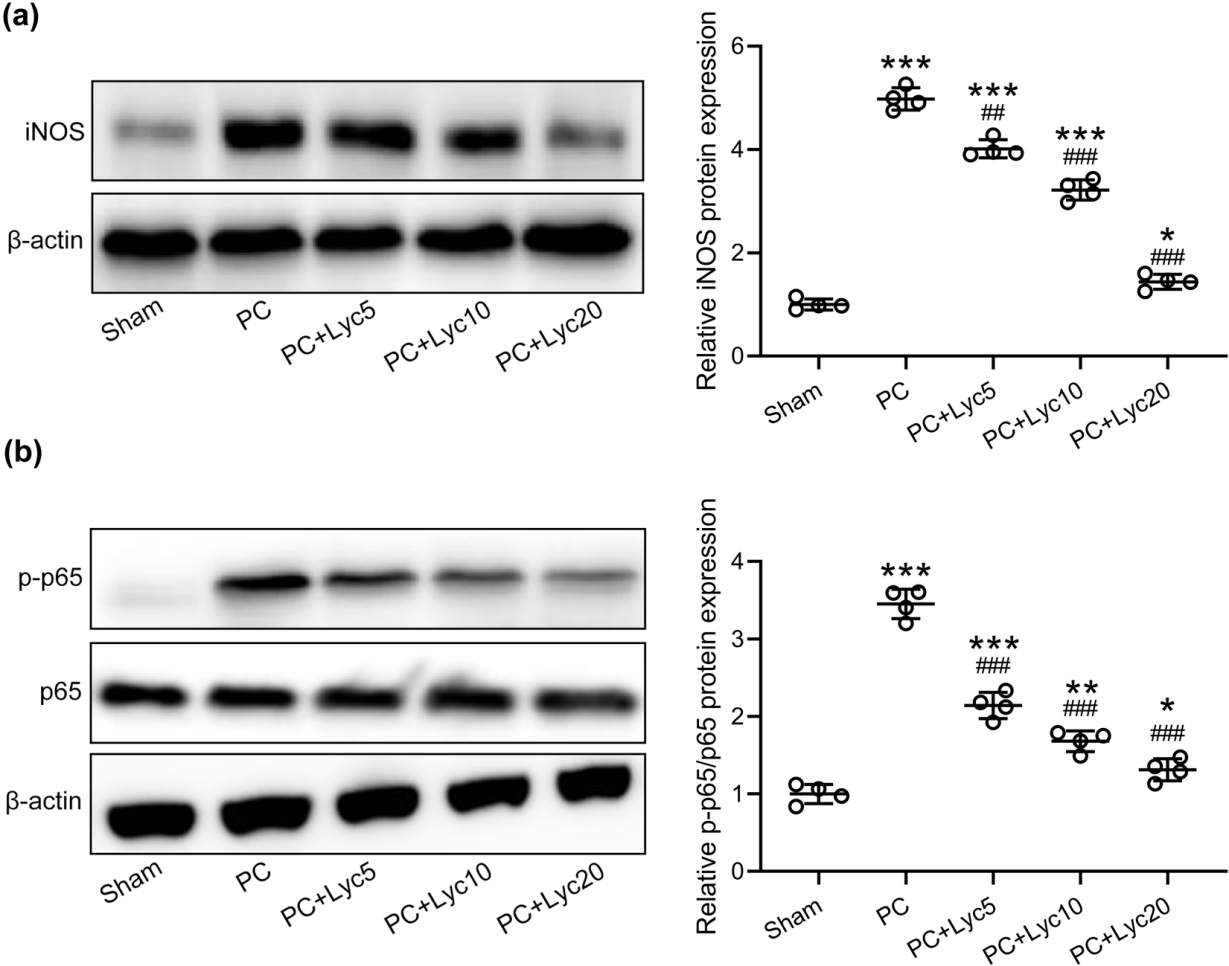
Effects of lycopene on iNOS expression and NF-κB activity in the bladder of rats with PC. (a) iNOS, (b) p-p65 and p65 levels in the bladder among different groups were analyzed by Western blot. *N* = 4 from ten mixed tissues for each group. Mean ± SD with all data presented. One-way ANOVA followed by Dunn’s multiple comparisons test. ^##^ *p* < 0.01, ^###^ *p* < 0.001 compared to the PC group. ^*^ *p* < 0.05, ^**^ *p* < 0.01, ^***^ *p* < 0.001 compared to the sham group.

In the PC group, the protein level of iNOS and the phosphorylation level of p65 subunit of NF-κB were significantly higher than in the other groups (Figure 6a and b). Lycopene treatment at doses of 5, 10, and 20 mg/kg/day was able to inhibit the expression of iNOS and the phosphorylation of p65.

## 4 Discussion

Previous studies have illustrated that rats with PC have increased bladder vascular permeability and decreased bladder blood flow [15,16]. Furthermore, another study has also demonstrated that the induction of PC increases the frequency of urination and urinary 8-OHdG level and decreases the interval between bladder contractions, locomotor activity, and urinary NO_*x*_ level [17].

In this research, we also established the rat PC model. Results showed that the induction of PC in rats decreased the interval between bladder contractions and the 24-h single-voided volume and increased the frequency of urination. However, lycopene administration reduced the frequency of urination and elevated the interval between bladder contractions and 24-h single-voided volume in PC rats. The effect of lycopene was also observed in a dose-dependent manner. Therefore, in the PC rat model, lycopene improved bladder overactivity and local vascular permeability.

Meanwhile, the induction of PC in rats decreased the locomotor activity and the urinary level of NO_*x*_ and increased the urinary level of 8-OHdG. Decreased locomotor activity suggests that PC-induced impairment is associated with pelvic pain or discomfort. Lycopene administration also enhanced locomotor activity. Meanwhile, the abnormal urinary 8-OHdG and NO_*x*_ levels in the PC rat model were also alleviated by lycopene. These results indicated that lycopene could elevate the urinary NO_*x*_ level. Nitric oxide has been shown to exhibit relaxant and facilitatory effects and act directly on bladder smooth muscle [18]. NO_*x*_ causes the relaxation of smooth muscle by activating soluble guanylate cyclase to produce cGMP. In rats with PC, the decreased urinary NO_*x*_ level indicates that the alteration of urinary frequency is related to bladder tissue hypoxia [17]. In this study, decreased urinary NO_*x*_ was reversed by the administration of lycopene. Thus, lycopene might improve bladder tissue hypoxia by relaxing the bladder and the pelvic vessels.

Lycopene contains several conjugated double bonds [19]. *In vivo*, lycopene is oxidized and degraded to form carbon chain-shorted isomers as metabolites, including 2,6-cyclolycopene-1 and 5,6-dihydroxy-5′, 6′-dihydrolycopene [20]. Lycopene acts as a free radical scavenger to prevent oxidative injury [21].

Increased urinary levels of oxidative stress markers in the PC rat model suggest that bladder tissue hypoxia is one of the factors contributing to lower urinary tract symptoms [16]. The high frequency of urination in a chronic bladder ischemia rat model is associated with increased oxidative stress in the bladder tissue [22]. 8-OHdG is an oxidative stress marker. PC decreases locomotor activity, increases the urinary 8-OHdG level, and decreases urinary NO_*x*_ [17]. Increased urinary 8-OHdG levels in the PC rat model were alleviated by lycopene. Meanwhile, the expression of IL-1β, IL-6, and iNOS in the bladder tissue was enhanced in PC rats. These results demonstrated that the inflammation response in the bladder was enhanced in rats with PC. The expression of IL-1β, IL-6, and iNOS was all inhibited by lycopene, which supports its potent anti-inflammatory effect. In the inflammatory response, IL‐1β is the vital pro-inflammatory factor and IL-6 is the crucial immune factor. Meanwhile, iNOS enhances oxidative stress by participating in extensive oxidative damage through the generation of NO and superoxide anions [23]. NO displays both relaxant and facilitatory effects on the bladder smooth muscle [18]. Decreased urinary NO_*x*_ in the PC rat model suggests that bladder tissue hypoxia is one of the causes of altered urinary frequency [17].

NF-κB regulates oxidative stress and inflammatory response. In the inactivated state, p65/p50 heterodimer binds to IκB and locates in the cytoplasm. In the activated state, IκB is degraded and the p65 subunit is phosphorylated and translocated into the nucleus to induce inflammatory factor expression [24,25]. NF-κB is an inducer of iNOS, which is normally absent in the bladder but is largely expressed during inflammatory conditions [26]. In mice with an overactive bladder profile, bladder inflammation and increased NF-kB/iNOS signaling were observed [26]. Studies have demonstrated the function of lycopene in the NF‐κB signaling pathway. Hung et al. have illustrated that lycopene affects the NF‐κB pathway to suppress tumor necrosis factor‐α-mediated expression of intercellular adhesion molecule‐1 [27]. Another research has indicated that the activation of NF‐κB is inhibited by lycopene [28]. Since lycopene showed a significant anti-inflammatory effect in the bladder of rats with PC, it might also regulate the NF‐κB pathway to alleviate the inflammation response.

Therefore, in this research, we also explored the effect of lycopene on the activity of the NF‐κB signaling pathway. Elevated iNOS protein levels in the bladder of the PC rat model indicated an enhanced inflammatory response. However, the administration of lycopene effectively decreased the iNOS protein level in the bladder of the PC rat model. In rats with PC, p65 phosphorylation in the bladder was significantly enhanced, but its total protein level remained unchanged, which showed enhanced NF‐κB pathway activity. Lycopene remarkably suppressed p65 phosphorylation but had no influence on total p65 protein level, which indicated that lycopene prevented the activation of NF‐κB.

In conclusion, the induction of PC in rats decreases locomotor activity, increases the frequency of urination, shortens the interval between bladder contractions, elevates urinary 8-OHdG level, and reduces urinary No_*x*_ level. All these symptoms could be effectively reversed by the administration of lycopene. Furthermore, in the bladder of rats with PC, pro-inflammatory mediator expression and the activity of the NF‐κB signaling pathway are all enhanced.

## Supplementary Material

Supplementary Figure
