# Supplementary material for: Lycopene ameliorates locomotor activity and urinary frequency induced by pelvic venous congestion in rats
Source: Open Med (Wars). 2023 Feb 25;18(1):20230638. doi: 10.1515/med-2023-0638 (PMC9971737; doi:10.1515/med-2023-0638)
Supplement: Supplementary Figure [file med-2023-0638-sm.pdf]

Supplementary material

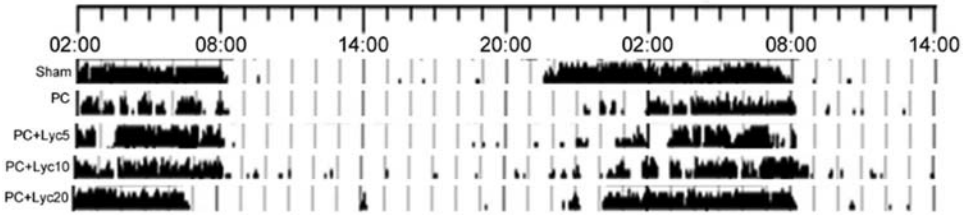

Figure S1: Representative actograms of locomotor activities among different groups.
